# The efficacy and safety of rituximab in treating childhood nephrotic syndrome: an Italian perspective

**DOI:** 10.1186/s13052-016-0271-6

**Published:** 2016-07-12

**Authors:** Dario Maratea, Monica Bettio, Maria Grazia Corti, Giovanni Montini, Francesca Venturini

**Affiliations:** Hospital Pharmacy, Fondazione IRCCS Ca’ Granda - Ospedale Maggiore Policlinico, Milano, Italy; School of Specialization in Hospital Pharmacy, School of Pharmacy, University of Milano, Milano, Italy; Pediatric Nephrology and Dialysis Unit, Department of Clinical Sciences and Community Health, University of Milan, Fondazione IRCCS Ca’ Granda - Ospedale Maggiore Policlinico, Milano, Italy

**Keywords:** Rituximab, Nephrotic syndrome, 648/96 law, Off label, Reimbursement

## Abstract

**Background:**

Nephrotic syndrome is a disorder characterized by proteinuria, hypoalbuminemia and dyslipidemia. Low-dose alternate-day steroid regimen is the standard of care. In case of relapse or significant adverse events, steroid-sparing agents may be used. This analysis was aimed at assessing the efficacy and safety of rituximab for the treatment of children with nephrotic syndrome.

**Results:**

Four studies were included in the final meta-analysis. The end-point of our analysis was the percentage of patients in remission at 6 months. Pooled data from the four studies favours the use of rituximab (RR 5.25, 95 % CI: 3.05–9.06; *p* < 0.0001). As regards the safety data, rituximab has a limited number of adverse effects, the most common of which occur during the infusions.

**Conclusions:**

In Italy, the off-label use of drugs is regulated by Law 648/96. In our opinion, there are three scientific requirements to merit a conditional national reimbursement for rituximab in nephrotic syndrome: 1. favourable clinical efficacy and safety data; 2. no available alternatives; 3. outcome data collecting by AIFA through prescribers. In conclusion, our results report a significant incremental benefit of adding rituximab to corticosteroid and/or calcineurin inhibitors for the treatment of nephrotic syndrome.

## Background

Nephrotic syndrome (NS) is a disorder characterized by heavy proteinuria, hypoalbuminemia (serum albumin <2.5 g/dl), often associated with dyslipidemia and hypercoagulability (Ravani et al. 2015). The most recent update of the NS clinical guidelines suggests a low-dose alternate-day steroid regimen as first-line treatment for the management of children who develop frequently-relapsing (FRNS) or steroid-dependent nephrotic syndrome (SDNS) [1].

However, when there is a failure to maintain remission or significant adverse events occur with corticosteroid therapy, clinicians have the option of using a number of steroid-sparing agents such as cyclophosphamide and calcineurin inhibitors (CIs, e.g., cyclosporin, tacrolimus, mycophenolate mofetil) or levamisole. Some of these immunosuppressant agents may cause serious adverse events such as nephrotoxicity, hyperglycemia, headaches and dyslipidemia [2]. Therefore, new drugs are needed to address this problem. Several reports and guidelines have proposed rituximab as a novel agent for the treatment of children with FRNS/SDNS [1–4].

During an evaluation of the off-label prescription of drugs in our hospital, we encountered many prescriptions for rituximab for the treatment of children with NS. Thus, the aim of this analysis was to assess the efficacy and safety data of the use of rituximab for the treatment of children with FRNS/SDNS, in whom the corticosteroid therapy is not sufficient to manage the disease, and to provide these quantitative results as meta-analytical data.

### Efficacy and safety data

We performed a literature search in PubMed on 13 January, 2016, using the following search terms: nephrotic syndrome AND rituximab; limits, clinical trials. The search identified 21 articles. After screening the titles and the abstracts, 3 studies underwent full-text screening [5–7]. Any randomised controlled trial addressing effectiveness and/or safety of rituximab for children with complicated FRNS/SDNS is considered eligible to be included. The inclusion criteria were the following: a) children with complicated FRNS/SDNS; b) rituximab as intervention therapy; c) corticosteroid therapy and/or CIs as control therapy; d) complete remission rate as end-point. A fourth study (Ahn et al. 2013 [8]) was extrapolated from the references of the paper by Iijima et al. 2014. Thus, 4 studies were included in the final analysis (Table 1). The intervention group comprised patients treated with rituximab plus prednisone and/or CIs, while the control group contained patients on prednisone and/or CIs. The end-point of our meta-analysis was the percentage of patients in remission at 6 months. The data of other end-points (e.g., relapse-free survival rate for efficacy and adverse events for safety) were reported as qualitative results. Our meta-analysis was performed using the RevMan software (version 5.2, the Nordic Cochrane Centre, Copenhagen, Denmark).

**Table 1:** Basic characteristics of included studies. The intervention group comprised patients treated with rituximab plus prednisone and/or CIs, while the control group contained patients on prednisone and/or CIs. The end-point of our meta-analysis was the percentage of patients in remission at 6 months

First author, year (Reference)	Study design	Intervention group (*n*)	Control group (*n*)	Age intervention group/age control group, yr, mean	Male/Female patients, n/n	Intervention
Ravani et al. 2015 [6].	Single-centre, RCT	RTX (15)	Corticosteroid therapy (15)	6.9/6.9	19/11	All patients were maintained in remission with high prednisone doses (>0.7 mg/kg per day) Treatment group - RTX (one infusion of 375 mg/m2) - prednisone was tapered off by 0.3 mg/kg per week if proteinuria was <1 g/d. Control group - prednisone (mean dose 49 mg/m2 per day)
Ravani et al. 2011 [5]	Single-centre, RCT	RTX (27)	Corticosteroid + CIs therapy (27)	10.2/11.3	43/11	Treatment group - RTX (one or two infusion of 375 mg/m2) - chlorfenamine maleate - methyl prednisolone - paracetamol - prednisone was tapered off by 0.3 mg/kg per week if proteinuria was <1 g/d. Control group - prednisone and CIs (tapered off by 0.3 mg/kg per week if proteinuria was <1 g/d.)
Iijima et al. 2014 [7]	Multicentre, RCT	RTX (24)	Corticosteroid therapy (24)	11.5/13.6	34/14	Treatment group - RTX an intravenous dose of 375 mg/m2 (maximum 500 mg) once weekly for 4 weeks. - Methylprednisolone - Acetaminophen - d-chlorpheniramine maleate Control group - prednisolone (60 mg/m2 orally three times a day (maximum of 80 mg per day) for 4 weeks, and then tapered over 6 weeks.
Ahn et al. 2013 [8]	Multicentre, RCT	RTX (35)	Corticosteroid + CIs therapy (18)	13/NA	NA/NA	Treatment group - single dose of intravenous RTX (375 mg/m2) Control group - corticosteroid therapy

FRNS or SDNS were defined complicated as follows: a) children when aged 2 years or older, who had ≥4 relapses in a 12-month period or steroid dependence at any point in the 2 years before relapse at screening, after completion of immunosuppressive drug treatment (e.g., ciclosporin, cyclophosphamide, mizoribine, or mycophenolate mofetil); or b) children when aged 2 years or older, who had ≥4 relapses in a 12-month period or steroid dependence diagnosed at any point in the 2 years before relapse at screening, during immunosuppressive drug treatment (e.g., ciclosporin, cyclophosphamide, mizoribine, or mycophenolate mofetil); or c) patients with a history of SRNS and diagnosed with FRNS or SDNS when aged 2 years or older, who had ≥4 relapses in a 12-month period or steroid dependence at any point in the 2 years before relapse at screening, during or after the completion of immunosuppressive drug treatment (e.g., ciclosporin or a combination of ciclosporin and methylprednisolone)

*Abbreviations*: *CIs* calcineurin inhibitor, *RTX* rituximab, *NA* not available

**Fig. 1:**
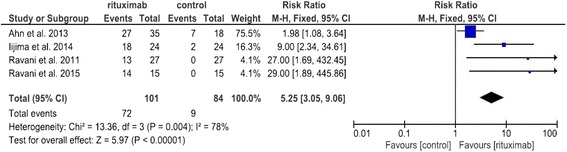
Forest plot showing a meta-analysis for rituximab treatment group versus control treatment group on complete remission rate at 6 months

Figure 1 shows the results of the meta-analysis. Pooled data from the four studies favours the use of rituximab as regards patients in remission at 6 months (RR 5.25, 95 % CI: 3.05–9.06; *p* < 0.0001). At 12 months, these results were also confirmed in two of the four above mentioned RCTs [5, 6]. The study by Iijima et al. 2014 reported that a median relapse-free survival rate favoured rituximab vs. control therapy (HR 0.27, 95 % CI: 0.14–0.53; *p* < 0.0001). The same results were reported by Ravani et al. 2014 (HR 0.39, 95 % CI: 0.22–0.67; *p* = 0.03). In the study by Ahn et al. 2014, these data were not available.

As regards the safety data, rituximab has a limited number of adverse effects, the most common of which occur during the infusions [5, 6]. In the study by Iijima et al. 2014, most adverse events for rituximab were mild, and no patient died during the trial. Although more patients in the rituximab group had serious adverse events compared to controls, the difference was not significant (*p* = 0.36). The most common grade 3–4 adverse events in the rituximab group were hypoproteinemia, lymphocytopenia and neutropenia.

Both studies by Ravani et al. report similar safety data, the most common adverse events being bronchospasm, hypotension (at the second rituximab infusion), skin rash, acute arthritis at the hip joint after 2 and 6 days from the infusion (resolution was rapidly and completely achieved within 24 to 48 h with non-steroidal anti-inflammatory medications). In the study by Ahn et al. 2014, 24 of the 54 treated patients (44 %) experienced mild and transient infusion reactions, however, no serious side effects were observed.

## Discussion

In Italy, the off-label use of drugs is currently regulated by Law 648/96. According to this regulation, medicines can be used off-label at NHS expense, once the Italian Medicine Agency (Agenzia Italiana del Farmaco, AIFA [9]) has authorised their inclusion on a specific list. The inclusion on this list requires the coexistence of three elements: favourable clinical efficacy and safety data; no or scant alternatives for treating the disease; outcome data collection by AIFA through prescribers. In our opinion, all the above mentioned requirements are met to merit a conditional national reimbursement for rituximab in NS through the law 648/96. However, the third requirement (e.g., collection of outcome data) should be made more stringent by AIFA and, in this case, it would allow for a pharmaco-epidemiological description of the treatments performed nationwide, compared to the current situation in which each individual hospital manages and analyses its own small pool of patients. The cost of one infusion of rituximab (375 mg/m2) is 1,943 euros/patient (this cost does not take into account any eventual nationally-negotiated procurement discount).

A new humanized anti-CD20 antibody - ofatumumab - has been developed and is currently being tested in two clinical trials: 1. Ofatumumab vs rituximab for children with SDNS (trial identifier NCT02394106 [10]); 2. ofatumumab vs placebo for children with FRNS (Basu 2014; Bonanni et al. 2015; trial identifier NCT02394119 [11–13]). The results are expected in the coming years; therefore, to date, rituximab is the best available alternative therapy to corticosteroids and/or CIs. The cost for one infusion of ofatumumab (1500 mg/m2) is 6,268 euros/patient (this cost does not take into account any eventual nationally-negotiated procurement discount).

The important aspects related to the price and the costs of these two monoclonal antibodies need to be taken into consideration. On one hand, rituximab is a well-known monoclonal antibody that became off-patent in Europe in November 2013 [14], although it is not yet marketed as such; on the other hand, ofatumumab is a new monoclonal antibody with a hypothetical future conditional approval for the treatment of children with NS, which costs more and, up to now, has less evidence supporting its use than rituximab does. In other words, to date, the reimbursement of rituximab under Law 648/96 might represent a cost-saving opportunity for the NHS to provide a treatment option for children with complicated FRNS/SDNS, in spite of the limited favourable supporting evidence available, at a lower price than ofatumumab, in case both drugs are included on the 648/96 list.

## Conclusion

The results of our updated meta-analysis report a significant incremental benefit of adding rituximab to corticosteroids and/or CIs when treating children with complicated FRNS/SDNS.
